# Supplementary material for: Uncovering the subtype-specific temporal order of cancer pathway dysregulation
Source: PLoS Comput Biol. 2019 Nov 11;15(11):e1007451. doi: 10.1371/journal.pcbi.1007451 (PMC6872169; doi:10.1371/journal.pcbi.1007451)
Supplement: Attachment — Submitted filename: ResponseReviewer2.docx [file pcbi.1007451.s001.docx]

**We appreciate the reviewer’s constructive feedback and provide our responses in bold format below each comment in the following. The changes are highlighted in the manuscript by red.**

***Reviewer #2:***

Building on a previously established Integer Linear program (PLPM) (see ref. [1]), in this study Khakabimamghani et al. have introduced a new method, called SPM to address the reconstruction of the temporal order of pathway dysregulation. They have extended the previous work (see ref. [1]) by using CCF values instead of binarized data and also, they have taken the subtype specificity of cancer progression into account in their formulation of the integer linear program. While their focus on the subtype-specificity of cancer progression is much appreciated, I have major issues with the originality/novelty of their methodology and the solidity of their results. Therefore, although I do not see the potential to recommend this work for publication in PLOS Computational Biology, I am pretty sure that it may warrant publication in an alternative journal (e.g. PLOS One), provided the major concerns are adequately addressed.

*Major comments:*

Comment #1: I appreciate the authors attempt to include subtype-specificity of cancer progression in reconstruction of the ordering of cancer pathway dysregulation, which has resulted in some improvements (particularly in the simulation results) as compared to the previous study (see ref [1]). However, I have the impression that their framework is very similar to the previous work and so to my view, it is not sufficiently novel.

**We recognize the reviewers perspective, however, we disagree based on the following. The previous method, PLPM, incorporates the concepts of mutual exclusivity and pathway-based progression order. We borrow these concepts and introduce two new and important biological aspects including subtype-specificity of disease progression and the relationship between mutation timing and its prevalence quantified through CCF values. Therefore, we provide important conceptual contributions that significantly changes the problem definition.**

**In terms of the methodology used for solving the problem, integer linear programming is a natural first choice for an NP-Hard problem and since there are two fundamental concepts (i.e. mutual exclusivity and pathway-based progression) common between the problem definitions for PLPM and SPM, we borrow the general framework used in PLPM. However, despite the conceptual commonalities, there are major non-trivial differences between the two methods. Introduction of subtype-specificity and non-binary CCF values as a proxy for mutation time requires using more complex variables (e.g. a three-dimensional variable) as well as a different objective function and novel constraints that require conversion from their natural quadratic form to linear form in order to make the model feasible. These additions and changes that we make to solve a different problem, SPM, are non-trivial, new and significantly different with the PLPM model. The proof of NP-hardness also requires modifications that are non-trivial.**

**Based on these significant differences, we believe that the proposed method qualifies as a novel contribution and we ask the reviewer to reevaluate the proposed model in light of the above explanations.**

Moreover, as the authors have clearly acknowledged in the conclusion:

“Due to the complexity of SPM, finding the optimal solution is hindered by factors such as quality of the input data and available computational power. This also restricts the number of genes and samples that can be included in SPM’s input. Therefore, employing alternative optimization methods is a new direction for future work.”

the method seems to suffer from complexity issues that is not well-addressed in this study.

**Although the above sentences only describe the complexity of SPM, which is an NP-Hard problem, we note that the comparison partner, i.e. PLPM, which is also NP-Hard, suffers from complexity issues too and the above sentences apply to that method as well. As an alternative method that produces more outputs, SPM still can yield meaningful results in a reasonable amount of time as described in the experimental results section.**

**We revised the above sentences as below to reflect this fact better:**

**“As an NP-Hard problem, similar to PLPM, the ILP solution for SPM is hindered by factors such as quality of the input data and available computational power. This also restricts the number of genes and samples that can be included in the input. Therefore, employing alternative optimization methods is a new direction for future work.”**

Comment #2: Although based on their sensitivity analysis using synthetic data, the authors have shown that their method more accurately assigns genes to pathways and more consistently reconstructs their ordering (as summarized in figure 2), their analysis of real dataset, which is exactly the same as the ones used in the previous study (see ref [1]), does not provide conclusive evidence regarding the advantage of including the subtype-specificity of cancer progression. This is acknowledged by the authors in multiple paragraphs in their Real Data Results section:

“Where SPM struggles is in grouping the PI3K pathway genes together, spreading them across identified pathways. This could potentially be due to the solution being sub-optimal as a result of insufficient data and model complexity”

“The separation of genes into their known biological pathways, as defined in [43], is less clear with the COAD dataset, with genes being spread across the identified pathways.”

“There is no clear advantage of any of the four identified progressions compared to the progression from PLMP, as they all struggle to fully recapitulate the true pathways, and are fairly similar, in regards to their identified pathways.”

“The resulting subtypes for COAD failed to show any meaningful difference in the survival analysis, unlike the GBM results.”

**We compare SPM with PLPM with respect to detected pathways (based on known pathways and the violation of mutual exclusivity) and evaluate the subtypes identified by SPM (with respect to survival and disease stages). We have discussed the strengths and weakness for both methods. For example, the first quoted sentence above indicates possible failure of SPM in only one out of four detected pathways (i.e. three of the pathways are meaningful), whereas PLPM, despite the premise of the method, finds two large pathways that mix a wide functional variety of genes that violate mutual exclusivity considerably (in ~14% of cases). Moreover, the last quoted sentence above indicates that the identified subtypes does not correspond to different survivals, but, according to figure 3(b) and as discussed in the manuscript, these subtypes distinguish different disease stages, which is a meaningful result.**

**Although we try to be honest about the limitations of SPM, the method has a number of strengths in comparison to PLPM such as identifying meaningful subtypes (which cannot be found using PLPM) as well as finding at least equally reasonable and often more informative pathways compared to PLPM (with respect to known pathways and mutual exclusivity). Despite the described weaknesses, many of which are shared by PLPM, the superiority of SPM becomes clearer in both synthetic and real-world data analysis.**

**Moreover, we are evaluating the truth of the identified pathways against ‘known’ pathways. However, these ‘known’ pathways are potentially ambiguous and incomplete, i.e. we don’t necessarily know the extent of all interactions between proteins in a cell and where we draw the boundary between pathways can be potentially arbitrary. So, the ground truth in this case should be treated with some skepticism. Given that SPM identifies meaningful strata, perhaps the pathways found by SPM can be informative and generate hypotheses about new interactions in a cell even if they don’t match the ‘known’ pathways exactly.**

**We made the following changes in the text to make the above distinctions clearer. We removed the below mostly speculative sentence:**

**“This could potentially be due to the solution being sub-optimal as a result of insufficient data and model complexity.”**

**We, also, made the following modification:**

**“Although we could not recognize any advantage for any of the four identified progressions compared to the progression from PLPM and no single progression fully recapitulating the known pathways, they provide a meaningful way to stratify patients by correlating to disease subtype. Therefore, identifying the separate subtypes can still be informative, in particular for finding the proper ordering of mutations.”**

*Minor comments:*

Comment #3: In the problem definition, the authors mention that: “For loci with copy number variation (CNV), computation of CCF is very difficult and associated with uncertainty. Therefore, we do not consider loci with CNV regardless of occurrence of SNV in the locus”

Would the authors comment on what is the prevalence of loci with CNV? Completely ignoring loci with CNV might have the potential to strongly bias the entire analyses. I therefore recommend the authors to clearly discuss this major issue in the conclusion or discussion section and provide quantitative data to ensure that ignoring loci with CNV data does not strongly mislead the results.

**As discussed in the beginning of the “Real Data Results” section, we limit our analysis to the genes that are known to be relevant to the disease of interest. Importantly, we only include the patients that do not have any CNV in any of these genes. Therefore,** **we avoid the type of bias that can be caused by ignoring CNVs that indeed exist in a patient.**

**To address the reviewer’s comment, we added the following to the “Real Data Results” section:**

**“This filtering avoids the type of bias that can be caused by ignoring CNVs that indeed exist in a patient. Although this process reduces sample size and limits our biological conclusions to the relationships between SNVs, the implications are still interesting with respect to the cancer progression and involved mutations.”**

Comment #4: In page 4, variables tau (Γ) and Z are introduced respectively as: “Γ as the sum of CCFs of genes within pathway x and Z as the corresponding CCF value of pathway x for sample I based on the CCFs of the genes belonging to x (described later) ”. The language is very unclear and according to these definitions, these two variables are almost indistinguishable. I suspect that they are used with different notation in equation (2) in page 12, where apparently the first term (equivalent of tau (Γ)) represents the “observed” CCF profiles and the second term (equivalent of Z) is the CCF profile “predicted” by SPM. I strongly recommend using the same language and notations in both places to avoid major confusions.

**The variable Γ is the sum of CCFs of all genes in the pathway while Z refers to only one CCF value which is chosen for a pathway out of all genes belonging to that pathway. Considering the reviewers comment, we discarded the sentence including the two variables because the preceding sentences are clearly describing the objective and the formal definition increases complexity without contributing much information. Later in the manuscript all outputs are formalized consistently.**

Comment #5: In the real data analyses, the authors have used exactly the same dataset as in (see ref [1]), which is focused only on glioblastoma multiforme and colorectal cancer. To prove the superiority of their method, analyses of alternative real datasets (including different cancer types) are required. At least, analysis of an alternative colorectal cancer dataset (see ref [2]) is necessary, which can help the authors to see whether their conclusions regarding colorectal cancer is robust and reproducible in different datasets?

**Unfortunately, the dataset at ref [2] does not contain read count information necessary for computing CCFs. Moreover, given that the current version of the manuscript explains comprehensive synthetic analysis and experiments on two real datasets (also used in the PLPM paper) with meaningful patient strata and/or pathways, and based on the time constraints for submitting the revised manuscript, we do not consider conducting further experiments at this point.**

Comment #6: In page 8, it is mentioned that “However, based on an empirical test for one of the experiments (not shown) and optimality gaps of ~13% and 0% respectively for COAD and GBM datasets, the results are expected to be near optimal, if not optimal.”. Please include the (not shown) results at least in the supplementary section.

**Because the mentioned empirical measures were for experiments with smaller numbers of patient and gene groups compared with the solutions reported for COAD and GBM, we decided to report the optimality gaps provided by CPLEX for the reported solutions, which accidentally match the previously mentioned values in this case. Therefore, the text is modified as follows:**

**“However, the optimality gaps computed by CPLEX for the presented results, which were respectively ~13% and ~0% for COAD and GBM datasets, indicate that the solutions are near optimal.”**

Comment #7: Please provide a more informative caption for the figure 4.

**The figure has two sub-figures each with its own caption. We separated the sub-figures as part of the response to comment #9 and used their own captions.**

Comment #8: In page 9, it is mentioned that “Additionally, all detected subtypes map well (Rand index =0.68) onto the five subtypes of GBM described in [52].”

It would be helpful, if the authors include a measure of the significance of the Rand index, for example the p-value quantified by cc_test_ari function in CrossClustering R package (see ref [3]).

**We appreciate the comment. In order to have a better picture of which clinical subtypes are identified by SPM, we replaced Rand index analysis with a pair-wise analysis between each detected subtype and each clinical subtype using Jaccard index. We reported the results in the manuscript as below:**

**“Additionally, two of the detected subtypes map reasonably onto two of the five subtypes of GBM described in [52]. Subtype 4 captures the G-CIMP subtype with Jaccard index 0.47 (FDR p-value 0.04) and subtype 2 maps to Neural subtype with Jaccard index 0.33 (FDR p-value 0.07).”**

Comment #9: In page 9 it is written “… and thus drugs that target early mutations should be more effective”. The logic behind this conclusion is not easy to follow, instead it seems to be highly speculative. I recommend avoiding such sentences at least in the results section.

**The sentence is removed.**

Comment #10: In the main text, Figure 4b is discussed after figure 5. The reordering of the figures accordingly may be required.

**We appreciate this comment. The figures are reordered to match their text appearance order. Fig 4 is now showing the survival analysis for GBM subtypes and Figure 7 is added to show the relationship between COAD subtypes and stages.**

Comment #11: In the conclusions section in page 12, the authors conclude that
“Based on experiments with two real datasets, namely GBM and COAD, the detected subtypes correlated to the clinical attributes such as survival and tumor stage.”

I realized that this conclusion is untrue as it contradicts the author’s own results, which was previously described as:

“The resulting subtypes for COAD failed to show any meaningful difference in the survival analysis, unlike the GBM results.”.

**As discussed before, although the COAD subtypes do not correlate with survival, but they are indicative of disease stage as shown in figure 4b. Therefore, the conclusion is valid.**

Comment #12: In the conclusions section in page 12, the authors conclude that “The relative importance of the pathways in different subtypes with respect to the progression models was consistent with the existing knowledge.”

I realized that this conclusion is also untrue as it contradicts the author’s own results, which was previously described as:

“The separation of genes into their known biological pathways, as defined in [43], is less clear with the COAD dataset, with genes being spread across the identified pathways.”
“There is no clear advantage of any of the four identified progressions compared to the progression from PLMP, as they all struggle to fully recapitulate the true pathways, and are fairly similar, in regards to their identified pathways.”

**The quoted sentence does not concern the validity of the pathways, but the order of the mutations within the detected pathways for different subtypes. We provide evidence for this conclusion, such as:**

**“In particular, both G-CIMP-high and our subtype 4 are characterized by a high frequency of mutations in IDH1, and in our case is the first mutation to occur (Fig 5).”**

**“Raphael and Vandin [47] made the argument that TP53 mutations occur before KRAS mutations, in contrast to previous reports for COAD but in line with more recent work on other cancer types. Although their progression supported this hypothesis, it is in conflict with the high CCF values for KRAS in some samples (Fig 5). In 88 cases (~67%), the progression from PLPM does not match the ordering of the CCF values, whereas SPM does not suffer from this issue due to its hard constraints. Our resulting subtypes are in agreement with the CCF values and suggest that the ordering of KRAS and TP53 is perhaps more variable than once thought, …”**

Comment #13: The last sentence of the conclusions in page 13 is too general and to my view is far beyond the scope of what this work has achieved: “The proposed framework provides a basis for gaining insight into the biology of cancer and its treatment as sequencing technologies advance, the quality of data increases, and larger datasets become available”.

**We appreciate the reviewer’s perspective. The sentence is eliminated.**

*References:*

[1] Raphael BJ, Vandin F. Simultaneous inference of Cancer Pathways and Tumor Progression from Cross-Sectional Mutation Data. In: Sharan R, editor. Research in Computational Molecular Biology. Cham: Springer International Publishing; 2014. P. 250-264.

[2] Wood L.D., Parsons D.W., Jones S., et al. 2007. The genomic landscapes of human breast and colorectal cancers. Science 318, 1108–1113.

[3] E.M. Qannari, P. Courcoux and Faye P. (2014) Significance test of the adjusted Rand index. Appli-cation to the free sorting task, Food Quality and Preference, (32)93-97
